# Supplementary material for: The Effect of Childhood Psychological Abuse on Depressive Symptoms in Adolescents Exposed to Campus Suicide: The Chain Mediating Role of Psychological Trauma and Anxiety Symptoms
Source: Behav Sci (Basel). 2025 Nov 20;15(11):1595. doi: 10.3390/bs15111595 (PMC12649577; doi:10.3390/bs15111595)
Supplement: Supplementary file 1 [file behavsci-15-01595-s001.zip › behavsci-3951456-supplementary.pdf]

Supplementary Materials

Table S1. T-test for different gender and only child groups 2

Table S2. Model summary information for models A1, A2, and A3 3

Table S3. Model summary information for models B1, B2, and B3 5

Table S4. Total, direct, and indirect effects for models A1, A2, B1, and B2 7

Figure S1. Path diagrams for the simple mediation models A1 and B1 8

Figure S2. Path diagrams for the simple mediation models A2 and B2 9

Table S5. Model summary information for models A1, A2, and A3 (Bootstrap) 10

Table S6. Model summary information for models B1, B2, and B3 (Bootstrap) 12

Table S7. Model summary information for models A2M, A2F, B2M, and B2F 14

Table S8. Model summary information for models A3M, A3F, B3M, and B3F 16

Table S9. Total, direct, and indirect effects for models A2M, A2F, B2M, and B2F ... 18

Table S10. Total, direct, and indirect effects for models A3M, A3F, B3M, and B3F . 19

Figure S3. Path diagrams for the simple mediation models A2M, A2F, B2M, and B2F 21

Figure S4. Path diagrams for the chain mediation models A3M, A3F, B3M, and B3F 23

Table S11. Model summary information for models A2M, A2F, B2M, and B2F (Bootstrap) 25

Table S12. Model summary information for models A3M, A3F, B3M, and B3F (Bootstrap) 27

Table S1. T-test for different gender and only child groups

Variable		CPA	PT	AS	DS
Gender	Male	18.37 ± 13.97	28.51 ± 17.65	3.94 ± 4.73	5.43 ± 4.99
	Female	17.91 ± 13.90	30.23 ± 18.22	4.40 ± 4.64	6.11 ± 4.91
	t	0.654	-1.926	-1.975^{*}	-2.738^{**}
Only child	Yes	17.79 ± 13.69	28.93 ± 17.55	4.12 ± 4.68	5.62 ± 4.90
	No	18.93 ± 14.43	30.32 ± 18.77	4.29 ± 4.72	6.09 ± 5.08
	t	-1.522	-1.412	-0.697	-1.748

Note. CPA: Childhood Psychological Abuse; PT: Psychological Trauma; AS: Anxiety Symptoms; DS: Depressive Symptoms.

N_{Male} = 806; N_{Female} = 797.

N_{Yes} = 1100; N_{No} = 503.

$^{*}p < 0.05$; $^{**}p < 0.01$ (two-tailed).

Table S2. Model summary information for models A1, A2, and A3

Model	Path	*b*	*SE*	β	*t*	95% CI (LL/ UL)
Model A1	CPA → PT	0.656	0.028	0.509	23.732***	0.602/ 0.710
	Age → PT	0.612	0.256	0.051	2.390*	0.110/ 1.114
	Gender → PT	1.970	0.770	0.055	2.557*	0.459/ 3.481
	CPA → DS	0.113	0.007	0.318	16.358***	0.100/ 0.127
	PT → DS	0.144	0.005	0.521	26.747***	0.134/ 0.155
	Age → DS	0.213	0.055	0.065	3.854***	0.105/ 0.321
	Gender → DS	0.461	0.166	0.047	2.772**	0.135/ 0.788
Model A2	CPA → PT	0.316	0.026	0.245	12.034***	0.264/ 0.367
	AS → PT	2.093	0.078	0.547	26.804***	1.940/ 2.246
	Age → PT	0.381	0.213	0.032	1.792	-0.036/ 0.799
	Gender → PT	0.868	0.641	0.024	1.353	-0.390/ 2.126
	CPA → DS	0.079	0.006	0.221	13.185***	0.067/ 0.090
	PT → DS	0.066	0.005	0.237	12.040***	0.055/ 0.076
	AS → DS	0.530	0.021	0.501	25.903***	0.490/ 0.570
	Age → DS	0.203	0.046	0.062	4.369***	0.112/ 0.294
	Gender → DS	0.337	0.140	0.034	2.411*	0.063/ 0.611
Model A3	CPA → PT	0.656	0.028	0.509	23.732***	0.602/ 0.710
	Age → PT	0.612	0.256	0.051	2.390*	0.110/ 1.114
	Gender → PT	1.970	0.770	0.055	2.557*	0.459/ 3.481
	CPA → AS	0.065	0.007	0.194	9.195***	0.051/ 0.079
	PT → AS	0.148	0.006	0.567	26.804***	0.137/ 0.159
	Age → AS	0.020	0.057	0.006	0.343	-0.092/ 0.131
	Gender → AS	0.235	0.171	0.025	1.374	-0.100/ 0.569
	CPA → DS	0.079	0.006	0.221	13.185***	0.067/ 0.090
	PT → DS	0.066	0.005	0.237	12.040***	0.055/ 0.076
	AS → DS	0.530	0.021	0.501	25.903***	0.490/ 0.570
	Age → DS	0.203	0.046	0.062	4.369***	0.112/ 0.294
	Gender → DS	0.337	0.140	0.034	2.411*	0.063/ 0.611

Note. CPA: Childhood Psychological Abuse; PT: Psychological Trauma; AS: Anxiety Symptoms; DS: Depressive Symptoms.

Model A1: Simple mediation model, controlling for age and gender; Model A2:

Simple mediation model, controlling for anxiety symptoms, age, and gender; Model A3: Chain mediation model, controlling for age and gender.

b: Unstandardized coefficient; β: Standardized coefficient.

$^{*}p < 0.05$, $^{**}p < 0.01$, $^{***}p < 0.001$ (two-tailed).

Table S3. Model summary information for models B1, B2, and B3

Model	Path	*b*	*SE*	β	*t*	95% CI (LL/ UL)
Model B1	CPA → PT	0.656	0.028	0.509	23.732***	0.602/ 0.710
	Age → PT	0.612	0.256	0.051	2.390*	0.110/ 1.114
	Gender → PT	1.970	0.770	0.055	2.557*	0.459/ 3.481
	CPA → AS	0.065	0.007	0.194	9.195***	0.051/ 0.079
	PT → AS	0.148	0.006	0.567	26.804***	0.137/ 0.159
	Age → AS	0.020	0.057	0.006	0.343	-0.092/ 0.131
	Gender → AS	0.235	0.171	0.025	1.374	-0.100/ 0.569
Model B2	CPA → PT	0.210	0.028	0.163	7.398***	0.154/ 0.266
	DS → PT	2.145	0.080	0.593	26.747***	1.988/ 2.303
	Age → PT	-0.034	0.214	-0.003	-0.160	-0.454/ 0.386
	Gender → PT	0.371	0.643	0.010	0.577	-0.891/ 1.633
	CPA → AS	0.002	0.006	0.006	0.329	-0.011/ 0.015
	PT → AS	0.068	0.006	0.259	12.135***	0.057/ 0.079
	DS → AS	0.558	0.022	0.590	25.903***	0.516/ 0.600
	Age → AS	-0.099	0.048	-0.032	-2.079*	-0.193/ -0.006
	Gender → AS	-0.023	0.144	-0.002	-0.159	-0.305/ 0.259
Model B3	CPA → PT	0.656	0.028	0.509	23.732***	0.602/ 0.710
	Age → PT	0.612	0.256	0.051	2.390*	0.110/ 1.114
	Gender → PT	1.970	0.770	0.055	2.557*	0.459/ 3.481
	CPA → DS	0.113	0.007	0.318	16.358***	0.100/ 0.127
	PT → DS	0.144	0.005	0.521	26.747***	0.134/ 0.155
	Age → DS	0.213	0.055	0.065	3.854***	0.105/ 0.321
	Gender → DS	0.461	0.166	0.047	2.772**	0.135/ 0.788
	CPA → AS	0.002	0.006	0.006	0.329	-0.011/ 0.015
	PT → AS	0.068	0.006	0.259	12.135***	0.057/ 0.079
	DS → AS	0.558	0.022	0.590	25.903***	0.516/ 0.600
	Age → AS	-0.099	0.048	-0.032	-2.079*	-0.193/ -0.006
	Gender → AS	-0.023	0.144	-0.002	-0.159	-0.305/ 0.259

Note. CPA: Childhood Psychological Abuse; PT: Psychological Trauma; AS: Anxiety Symptoms; DS: Depressive Symptoms.

Model B1: Simple mediation model, controlling for age and gender; Model B2:

Simple mediation model, controlling for depressive symptoms, age, and gender;

Model B3: Chain mediation model, controlling for age and gender.

b: Unstandardized coefficient; β: Standardized coefficient.

$^{*}p < 0.05$, $^{**}p < 0.01$, $^{***}p < 0.001$ (two-tailed).

Table S4. Total, direct, and indirect effects for models A1, A2, B1, and B2

Model	Variable	Effect	LLCI	ULCI	Ratio
Model A1	Total effect	0.208	0.194	0.222	—
	Direct effect: CPA → DS	0.113	0.100	0.127	54.50%
	Ind: CPA → PT → DS	0.095	0.084	0.106	45.50%
Model A2	Total effect	0.099	0.088	0.111	—
	Direct effect: CPA → DS	0.079	0.067	0.090	79.18%
	Ind: CPA → PT → DS	0.021	0.016	0.026	20.82%
Model B1	Total effect	0.163	0.148	0.177	—
	Direct effect: CPA → AS	0.065	0.051	0.079	40.22%
	Ind: CPA → PT → AS	0.097	0.086	0.109	59.78%
Model B2	Total effect	0.016	0.003	0.029	—
	Direct effect: CPA → AS	0.002	-0.011	0.015	12.88%
	Ind: CPA → PT → AS	0.014	0.009	0.020	87.12%

Note. CPA: Childhood Psychological Abuse; PT: Psychological Trauma; AS: Anxiety Symptoms; DS: Depressive Symptoms.

Model A1: Simple mediation model, controlling for age and gender; Model A2: Simple mediation model, controlling for anxiety symptoms, age, and gender.

Model B1: Simple mediation model, controlling for age and gender; Model B2: Simple mediation model, controlling for depressive symptoms, age, and gender.

Ind: Indirect effect.

Unstandardized effects were presented.

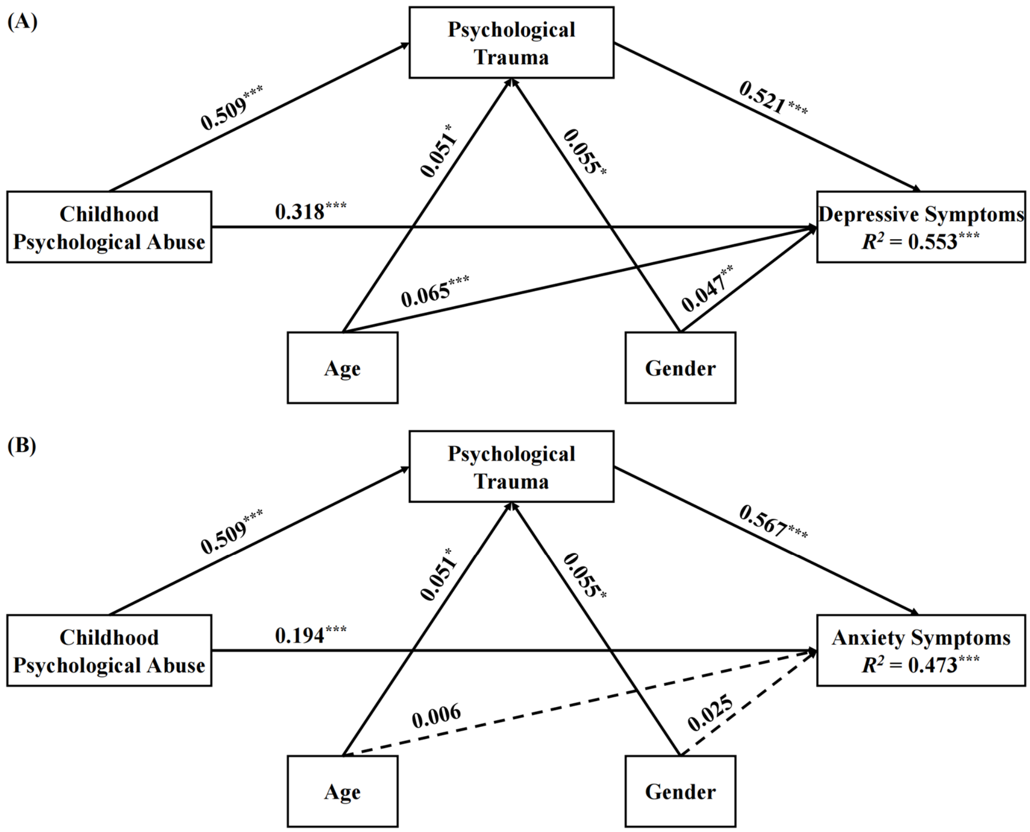

Figure S1. Path diagrams for the simple mediation models A1 and B1

Note: A: Model A1, B: Model B1.

Standardized coefficients were presented.

$^{*}p < 0.05$, $^{**}p < 0.01$, $^{***}p < 0.001$ (two-tailed).

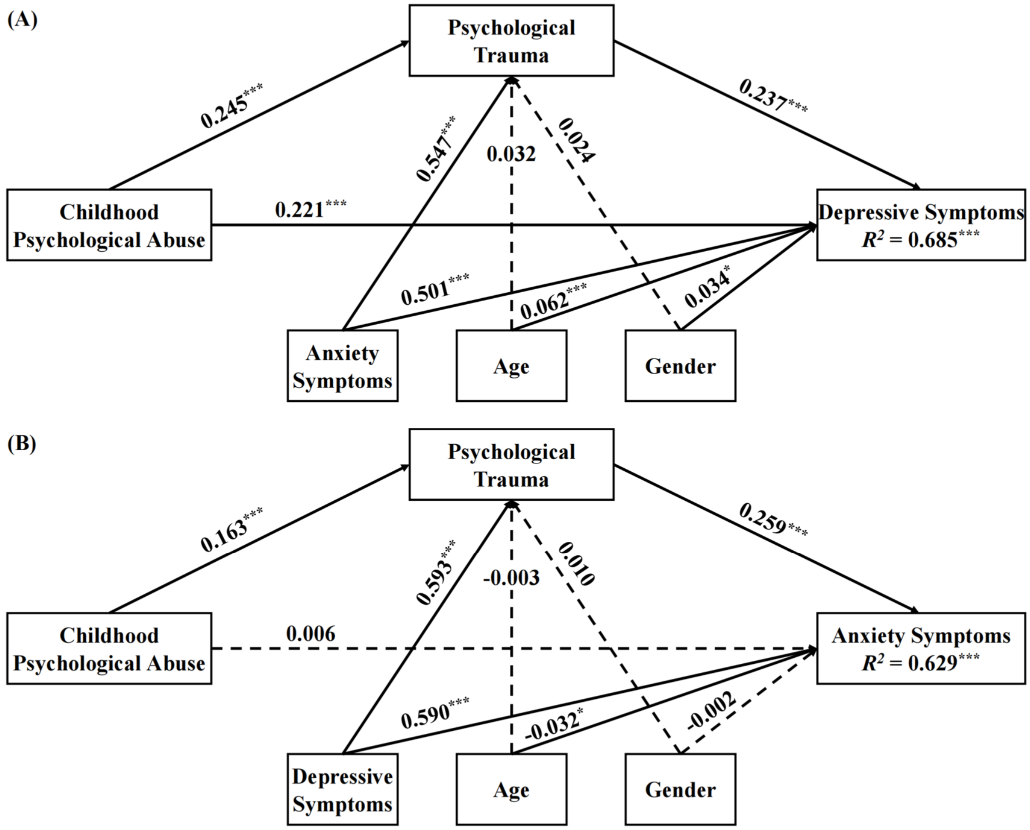

Figure S2. Path diagrams for the simple mediation models A2 and B2

Note: A: Model A2, B: Model B2.

Standardized coefficients were presented.

$^{*}p < 0.05$, $^{***}p < 0.001$ (two-tailed).

Table S5. Model summary information for models A1, A2, and A3 (Bootstrap)

Model	Path	Coeff	BootSE	BootLLCI	BootULCI
Model A1	CPA → PT	0.656	0.028	0.602	0.713
	Age → PT	0.612	0.248	0.137	1.106
	Gender → PT	1.970	0.759	0.448	3.426
	CPA → DS	0.113	0.009	0.096	0.130
	PT → DS	0.144	0.006	0.132	0.156
	Age → DS	0.213	0.055	0.103	0.322
	Gender → DS	0.461	0.164	0.149	0.783
Model A2	CPA → PT	0.316	0.028	0.261	0.371
	AS → PT	2.093	0.101	1.898	2.295
	Age → PT	0.381	0.214	-0.043	0.797
	Gender → PT	0.868	0.634	-0.376	2.095
	CPA → DS	0.079	0.008	0.064	0.094
	PT → DS	0.066	0.006	0.054	0.078
	AS → DS	0.530	0.027	0.476	0.584
	Age → DS	0.203	0.046	0.111	0.289
	Gender → DS	0.337	0.139	0.056	0.605
Model A3	CPA → PT	0.656	0.028	0.601	0.713
	Age → PT	0.612	0.249	0.117	1.093
	Gender → PT	1.970	0.767	0.459	3.471
	CPA → AS	0.065	0.009	0.047	0.084
	PT → AS	0.148	0.007	0.136	0.161
	Age → AS	0.020	0.054	-0.086	0.123
	Gender → AS	0.235	0.172	-0.103	0.569
	CPA → DS	0.079	0.008	0.064	0.093
	PT → DS	0.066	0.006	0.054	0.077
	AS → DS	0.530	0.027	0.479	0.583
	Age → DS	0.203	0.046	0.114	0.295
	Gender → DS	0.337	0.141	0.054	0.610

Note. CPA: Childhood Psychological Abuse; PT: Psychological Trauma; AS: Anxiety Symptoms; DS: Depressive Symptoms.

Model A1: Simple mediation model, controlling for age and gender; Model A2:

Simple mediation model, controlling for anxiety symptoms, age, and gender; Model A3: Chain mediation model, controlling for age and gender.

Bootstrap = 5000.

Table S6. Model summary information for models B1, B2, and B3 (Bootstrap)

Model	Path	Coeff	BootSE	BootLLCI	BootULCI
Model B1	CPA → PT	0.656	0.028	0.602	0.711
	Age → PT	0.612	0.250	0.129	1.104
	Gender → PT	1.970	0.765	0.496	3.499
	CPA → AS	0.065	0.009	0.048	0.084
	PT → AS	0.148	0.006	0.135	0.161
	Age → AS	0.020	0.055	-0.090	0.126
	Gender → AS	0.235	0.168	-0.094	0.561
Model B2	CPA → PT	0.210	0.033	0.146	0.275
	DS → PT	2.145	0.091	1.968	2.327
	Age → PT	-0.034	0.213	-0.452	0.389
	Gender → PT	0.371	0.648	-0.887	1.627
	CPA → AS	0.002	0.007	-0.012	0.017
	PT → AS	0.068	0.007	0.053	0.082
	DS → AS	0.558	0.030	0.499	0.617
	Age → AS	-0.099	0.046	-0.190	-0.008
	Gender → AS	-0.023	0.147	-0.307	0.267
Model B3	CPA → PT	0.656	0.029	0.601	0.712
	Age → PT	0.612	0.248	0.132	1.117
	Gender → PT	1.970	0.768	0.468	3.480
	CPA → DS	0.113	0.009	0.095	0.131
	PT → DS	0.144	0.006	0.132	0.156
	Age → DS	0.213	0.055	0.104	0.323
	Gender → DS	0.461	0.166	0.140	0.784
	CPA → AS	0.002	0.007	-0.012	0.016
	PT → AS	0.068	0.007	0.053	0.082
	DS → AS	0.558	0.030	0.500	0.617
	Age → AS	-0.099	0.045	-0.188	-0.010
	Gender → AS	-0.023	0.146	-0.311	0.266

Note. CPA: Childhood Psychological Abuse; PT: Psychological Trauma; AS: Anxiety Symptoms; DS: Depressive Symptoms.

Model B1: Simple mediation model, controlling for age and gender; Model B2:

Simple mediation model, controlling for depressive symptoms, age, and gender;
Model B3: Chain mediation model, controlling for age and gender.
Bootstrap = 5000.

Table S7. Model summary information for models A2M, A2F, B2M, and B2F

Model	Path	*b*	*SE*	β	*t*	95% CI (LL/ UL)
Model A2M	CPA → PT	0.313	0.037	0.247	8.452***	0.240/ 0.385
	AS → PT	1.945	0.109	0.522	17.842***	1.731/ 2.159
	Age → PT	0.857	0.310	0.071	2.761**	0.248/ 1.466
	CPA → DS	0.089	0.009	0.248	10.278***	0.072/ 0.106
	PT → DS	0.070	0.008	0.246	8.817***	0.054/ 0.085
	AS → DS	0.488	0.029	0.463	16.943***	0.432/ 0.545
	Age → DS	0.214	0.070	0.063	3.066**	0.077/ 0.351
Model A2F	CPA → PT	0.310	0.037	0.236	8.288***	0.236/ 0.383
	AS → PT	2.252	0.112	0.573	20.189***	2.033/ 2.471
	Age → PT	-0.026	0.293	-0.002	-0.088	-0.601/ 0.549
	CPA → DS	0.068	0.008	0.192	8.233***	0.052/ 0.084
	PT → DS	0.060	0.008	0.224	8.023***	0.046/ 0.075
	AS → DS	0.579	0.029	0.547	19.935***	0.522/ 0.636
	Age → DS	0.179	0.062	0.056	2.891**	0.058/ 0.301
Model B2M	CPA → PT	0.190	0.040	0.151	4.721***	0.111/ 0.270
	DS → PT	2.059	0.114	0.582	18.111***	1.836/ 2.283
	Age → PT	0.367	0.311	0.031	1.179	-0.244/ 0.978
	CPA → AS	0.003	0.010	0.007	0.255	-0.017/ 0.021
	PT → AS	0.070	0.008	0.261	8.379***	0.054/ 0.086
	DS → AS	0.540	0.032	0.570	16.943***	0.478/ 0.603
	Age → AS	-0.149	0.074	-0.046	-2.028*	-0.294/ -0.005
Model B2F	CPA → PT	0.226	0.040	0.172	5.629***	0.147/ 0.304
	DS → PT	2.229	0.113	0.601	19.731***	2.008/ 2.451
	Age → PT	-0.347	0.297	-0.029	-1.171	-0.929/ 0.235
	CPA → AS	0.003	0.009	0.008	0.322	-0.014/ 0.020
	PT → AS	0.066	0.007	0.258	8.811***	0.051/ 0.080
	DS → AS	0.577	0.029	0.611	19.935***	0.520/ 0.634
	Age → AS	-0.051	0.062	-0.017	-0.813	-0.173/ 0.072

Note. M: Male adolescents, N = 806; F: Female adolescents, N = 797.

CPA: Childhood Psychological Abuse; PT: Psychological Trauma; AS: Anxiety Symptoms; DS: Depressive Symptoms.

Model A2M & Model A2F: Simple mediation model, controlling for anxiety symptoms and age; Model B2M & Model B2F: Simple mediation model, controlling for depressive symptoms and age.

b: Unstandardized coefficient; β: Standardized coefficient.

$^{*}p < 0.05$, $^{**}p < 0.01$, $^{***}p < 0.001$ (two-tailed).

Table S8. Model summary information for models A3M, A3F, B3M, and B3F

Model	Path	*b*	*SE*	β	*t*	95% CI (LL/ UL)
Model A3M	CPA → PT	0.623	0.039	0.493	16.148***	0.547/ 0.698
	Age → PT	1.072	0.366	0.089	2.928**	0.353/ 1.791
	CPA → AS	0.068	0.010	0.202	6.649***	0.048/ 0.089
	PT → AS	0.146	0.008	0.545	17.842***	0.130/ 0.162
	Age → AS	-0.046	0.085	-0.014	-0.537	-0.214/ 0.122
	CPA → DS	0.089	0.009	0.248	10.278***	0.072/ 0.106
	PT → DS	0.070	0.008	0.246	8.817***	0.054/ 0.085
	AS → DS	0.488	0.029	0.463	16.943***	0.432/ 0.545
	Age → DS	0.214	0.070	0.063	3.066**	0.077/ 0.351
Model A3F	CPA → PT	0.683	0.040	0.521	17.127***	0.605/ 0.762
	Age → PT	0.232	0.360	0.020	0.643	-0.475/ 0.938
	CPA → AS	0.063	0.010	0.189	6.407***	0.044/ 0.082
	PT → AS	0.151	0.008	0.592	20.189***	0.136/ 0.165
	Age → AS	0.079	0.076	0.026	1.047	-0.069/ 0.228
	CPA → DS	0.068	0.008	0.192	8.233***	0.052/ 0.084
	PT → DS	0.060	0.008	0.224	8.023***	0.046/ 0.075
	AS → DS	0.579	0.029	0.547	19.935***	0.522/ 0.636
	Age → DS	0.179	0.062	0.056	2.891**	0.058/ 0.301
Model B3M	CPA → PT	0.623	0.039	0.493	16.148***	0.547/ 0.698
	Age → PT	1.072	0.366	0.089	2.928**	0.353/ 1.791
	CPA → DS	0.122	0.010	0.342	12.479***	0.103/ 0.141
	PT → DS	0.141	0.008	0.499	18.111***	0.126/ 0.156
	Age → DS	0.191	0.081	0.056	2.357*	0.032/ 0.351
	CPA → AS	0.003	0.010	0.007	0.255	-0.017/ 0.021
	PT → AS	0.070	0.008	0.261	8.379***	0.054/ 0.086
	DS → AS	0.540	0.032	0.570	16.943***	0.478/ 0.603
	Age → AS	-0.149	0.074	-0.046	-2.028*	-0.294/ -0.005
Model B3F	CPA → PT	0.683	0.040	0.521	17.127***	0.605/ 0.762
	Age → PT	0.232	0.360	0.020	0.643	-0.475/ 0.938
	CPA → DS	0.104	0.010	0.295	10.597***	0.085/ 0.124

PT → DS	0.148	0.008	0.548	19.731***	0.133/ 0.162
Age → DS	0.225	0.076	0.071	2.968**	0.076/ 0.375
CPA → AS	0.003	0.009	0.008	0.322	-0.014/ 0.020
PT → AS	0.066	0.007	0.258	8.811***	0.051/ 0.080
DS → AS	0.577	0.029	0.611	19.935***	0.520/ 0.634
Age → AS	-0.051	0.062	-0.017	-0.813	-0.173/ 0.072

Note. M: Male adolescents, N = 806; F: Female adolescents, N = 797.

CPA: Childhood Psychological Abuse; PT: Psychological Trauma; AS: Anxiety Symptoms; DS: Depressive Symptoms.

Model A3M & Model A3F: Chain mediation model, controlling for age; Model B3M & Model B3F: Chain mediation model, controlling for age.

b: Unstandardized coefficient; β: Standardized coefficient.

$^{*}p < 0.05$, $^{**}p < 0.01$, $^{***}p < 0.001$ (two-tailed).

Table S9. Total, direct, and indirect effects for models A2M, A2F, B2M, and B2F

Model	Variable	Effect	LLCI	ULCI	Ratio
Model A2M	Total effect	0.110	0.094	0.127	—
	Direct effect: CPA → DS	0.089	0.072	0.106	80.27%
	Ind: CPA → PT → DS	0.022	0.014	0.031	19.73%
Model A2F	Total effect	0.087	0.070	0.103	—
	Direct effect: CPA → DS	0.068	0.052	0.084	78.41%
	Ind: CPA → PT → DS	0.019	0.013	0.025	21.59%
Model B2M	Total effect	0.016	-0.004	0.035	—
	Direct effect: CPA → AS	0.003	-0.017	0.021	15.82%
	Ind: CPA → PT → AS	0.013	0.005	0.023	84.18%
Model B2F	Total effect	0.018	0.000	0.035	—
	Direct effect: CPA → AS	0.003	-0.014	0.020	15.91%
	Ind: CPA → PT → AS	0.015	0.009	0.022	84.09%

Note. M: Male adolescents, N = 806; F: Female adolescents, N = 797.

CPA: Childhood Psychological Abuse; PT: Psychological Trauma; AS: Anxiety Symptoms; DS: Depressive Symptoms.

Model A2M & Model A2F: Simple mediation model, controlling for anxiety symptoms and age; Model B2M & Model B2F: Simple mediation model, controlling for depressive symptoms and age.

Ind: Indirect effect.

Unstandardized effects were presented.

Table S10. Total, direct, and indirect effects for models A3M, A3F, B3M, and B3F

Model	Variable	Effect	LLCI	ULCI	Ratio
Model A3M	Total effect	0.210	0.190	0.230	—
	Direct effect: CPA → DS	0.089	0.072	0.106	42.26%
	Total indirect effect	0.121	0.103	0.139	57.74%
	Ind 1: CPA → PT → DS	0.043	0.032	0.056	20.63%
	Ind 2: CPA → AS → DS	0.033	0.019	0.049	15.91%
	Ind 3: CPA → PT → AS → DS	0.044	0.034	0.056	21.15%
	Compare 1: Ind 1 - Ind 2	0.010	-0.013	0.031	
	Compare 2: Ind 1 - Ind 3	-0.001	-0.019	0.015	
	Compare 3: Ind 2 - Ind 3	-0.011	-0.030	0.010	
Model A3F	Total effect	0.205	0.185	0.225	—
	Direct effect: CPA → DS	0.068	0.052	0.084	33.07%
	Total indirect effect	0.137	0.119	0.157	66.93%
	Ind 1: CPA → PT → DS	0.041	0.031	0.053	20.07%
	Ind 2: CPA → AS → DS	0.037	0.023	0.051	17.78%
	Ind 3: CPA → PT → AS → DS	0.060	0.049	0.071	29.08%
	Compare 1: Ind 1 - Ind 2	0.005	-0.015	0.024	
	Compare 2: Ind 1 - Ind 3	-0.019	-0.035	-0.002	
	Compare 3: Ind 2 - Ind 3	-0.023	-0.042	-0.005	
Model B3M	Total effect	0.159	0.139	0.180	—
	Direct effect: CPA → AS	0.003	-0.017	0.021	1.57%
	Total indirect effect	0.157	0.136	0.177	98.43%
	Ind 1: CPA → PT → AS	0.044	0.028	0.061	27.29%
	Ind 2: CPA → DS → AS	0.066	0.049	0.085	41.41%
	Ind 3: CPA → PT → DS → AS	0.047	0.036	0.060	29.74%
	Compare 1: Ind 1 - Ind 2	-0.023	-0.053	0.006	
	Compare 2: Ind 1 - Ind 3	-0.004	-0.026	0.019	
	Compare 3: Ind 2 - Ind 3	0.019	-0.001	0.039	
Model B3F	Total effect	0.166	0.146	0.186	—
	Direct effect: CPA → AS	0.003	-0.014	0.020	1.69%
	Total indirect effect	0.163	0.143	0.185	98.31%

Ind 1: CPA → PT → AS	0.045	0.032	0.058	26.99%
Ind 2: CPA → DS → AS	0.060	0.045	0.076	36.27%
Ind 3: CPA → PT → DS → AS	0.058	0.047	0.070	35.06%
Compare 1: Ind 1 - Ind 2	-0.015	-0.039	0.008	
Compare 2: Ind 1 - Ind 3	-0.013	-0.032	0.005	
Compare 3: Ind 2 - Ind 3	0.002	-0.017	0.020	

Note. M: Male adolescents, N = 806; F: Female adolescents, N = 797.

CPA: Childhood Psychological Abuse; PT: Psychological Trauma; AS: Anxiety Symptoms; DS: Depressive Symptoms.

Model A3M & Model A3F: Chain mediation model, controlling for age; Model B3M & Model B3F: Chain mediation model, controlling for age.

Ind: Indirect effect.

Unstandardized effects were presented.

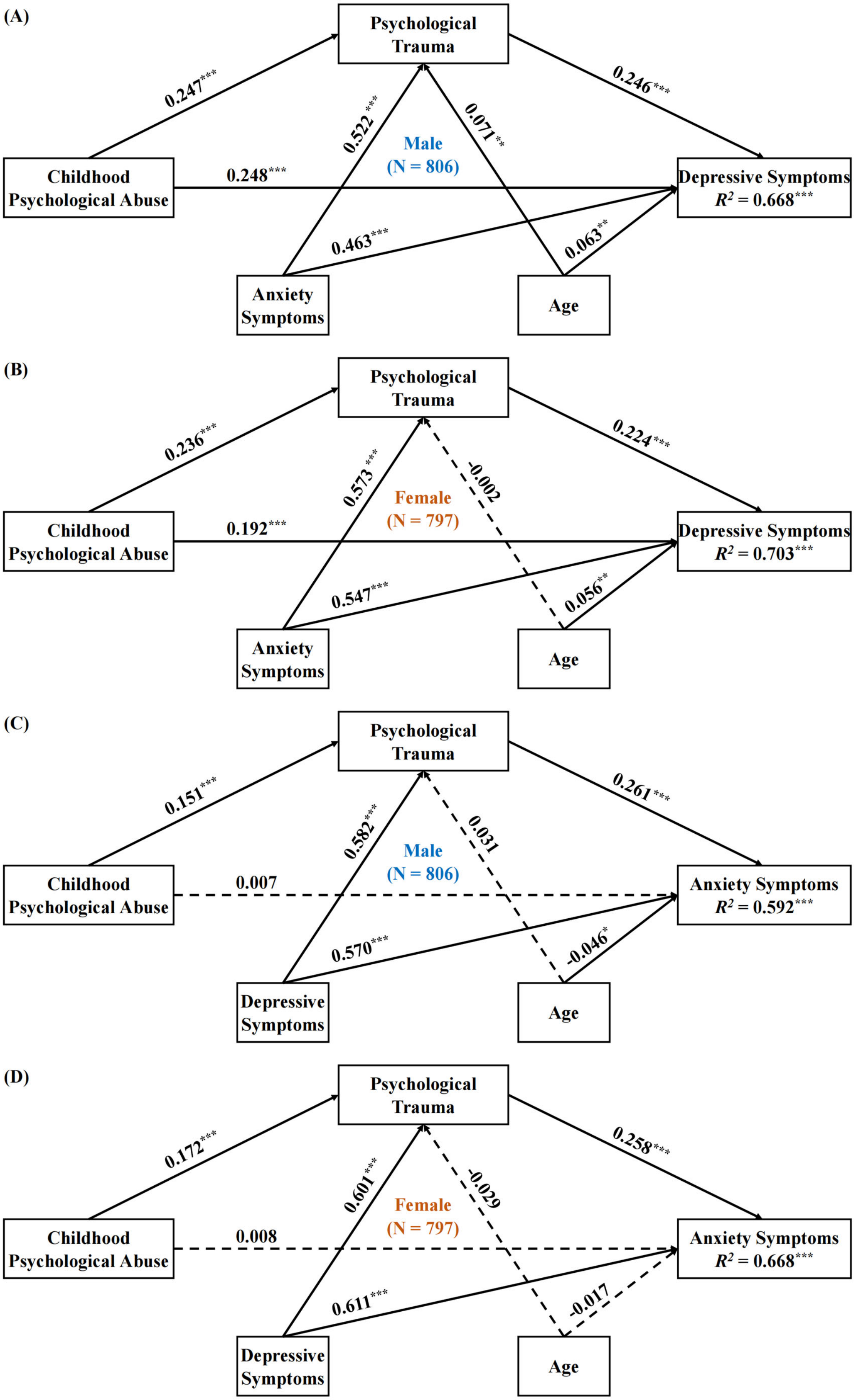

Figure S3. Path diagrams for the simple mediation models A2M, A2F, B2M, and

B2F

Note: A: Model A2M, B: Model A2F, C: Model B2M, D: Model B2F.

Standardized coefficients were presented.

$^{*}p < 0.05$, $^{**}p < 0.01$, $^{***}p < 0.001$ (two-tailed).

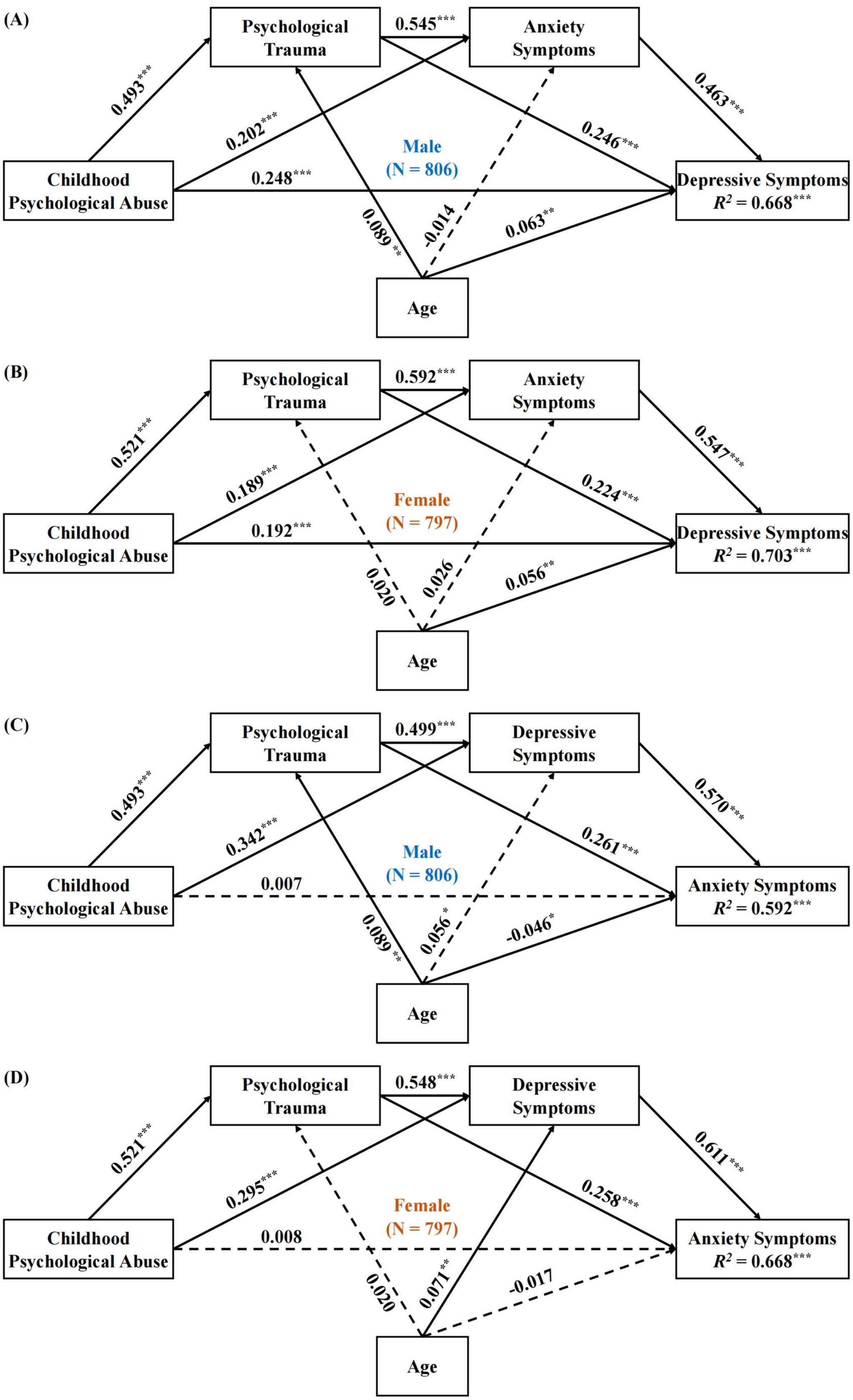

Figure S4. Path diagrams for the chain mediation models A3M, A3F, B3M, and B3F

Note: A: Model A3M, B: Model A3F, C: Model B3M, D: Model B3F.

Standardized coefficients were presented.

$^{*}p < 0.05$, $^{**}p < 0.01$, $^{***}p < 0.001$ (two-tailed).

Table S11. Model summary information for models A2M, A2F, B2M, and B2F (Bootstrap)

Model	Path	Coeff	BootSE	BootLLCI	BootULCI
Model A2M	CPA → PT	0.313	0.041	0.230	0.394
	AS → PT	1.945	0.144	1.670	2.237
	Age → PT	0.857	0.302	0.275	1.451
	CPA → DS	0.089	0.012	0.066	0.112
	PT → DS	0.070	0.009	0.052	0.087
	AS → DS	0.488	0.040	0.410	0.568
	Age → DS	0.214	0.071	0.078	0.353
Model A2F	CPA → PT	0.310	0.039	0.236	0.384
	AS → PT	2.252	0.134	1.985	2.513
	Age → PT	-0.026	0.289	-0.600	0.544
	CPA → DS	0.068	0.010	0.049	0.087
	PT → DS	0.060	0.008	0.045	0.076
	AS → DS	0.579	0.034	0.512	0.645
	Age → DS	0.179	0.060	0.059	0.296
Model B2M	CPA → PT	0.190	0.050	0.089	0.287
	DS → PT	2.059	0.135	1.803	2.327
	Age → PT	0.367	0.306	-0.233	0.965
	CPA → AS	0.003	0.011	-0.019	0.024
	PT → AS	0.070	0.012	0.047	0.092
	DS → AS	0.540	0.046	0.454	0.632
	Age → AS	-0.149	0.073	-0.293	-0.004
Model B2F	CPA → PT	0.226	0.043	0.143	0.311
	DS → PT	2.229	0.125	1.983	2.473
	Age → PT	-0.347	0.292	-0.916	0.230
	CPA → AS	0.003	0.010	-0.016	0.023
	PT → AS	0.066	0.009	0.048	0.083
	DS → AS	0.577	0.038	0.500	0.651
	Age → AS	-0.051	0.059	-0.162	0.069

Note. M: Male adolescents, N = 806; F: Female adolescents, N = 797.

CPA: Childhood Psychological Abuse; PT: Psychological Trauma; AS: Anxiety

Symptoms; DS: Depressive Symptoms.

Model A2M & Model A2F: Simple mediation model, controlling for anxiety symptoms and age; Model B2M & Model B2F: Simple mediation model, controlling for depressive symptoms and age.

Bootstrap = 5000.

Table S12. Model summary information for models A3M, A3F, B3M, and B3F (Bootstrap)

Model	Path	Coeff	BootSE	BootLLCI	BootULCI
Model A3M	CPA → PT	0.623	0.041	0.540	0.701
	Age → PT	1.072	0.346	0.385	1.753
	CPA → AS	0.068	0.014	0.041	0.098
	PT → AS	0.146	0.010	0.125	0.166
	Age → AS	-0.046	0.082	-0.206	0.119
	CPA → DS	0.089	0.012	0.065	0.111
	PT → DS	0.070	0.009	0.052	0.087
	AS → DS	0.488	0.040	0.411	0.570
	Age → DS	0.214	0.070	0.078	0.352
Model A3F	CPA → PT	0.683	0.040	0.605	0.762
	Age → PT	0.232	0.361	-0.467	0.950
	CPA → AS	0.063	0.012	0.040	0.086
	PT → AS	0.151	0.008	0.134	0.167
	Age → AS	0.079	0.074	-0.065	0.221
	CPA → DS	0.068	0.009	0.049	0.087
	PT → DS	0.060	0.008	0.046	0.075
	AS → DS	0.579	0.034	0.514	0.646
	Age → DS	0.179	0.059	0.067	0.297
Model B3M	CPA → PT	0.623	0.040	0.541	0.701
	Age → PT	1.072	0.344	0.405	1.744
	CPA → DS	0.122	0.013	0.096	0.148
	PT → DS	0.141	0.009	0.122	0.159
	Age → DS	0.191	0.082	0.028	0.355
	CPA → AS	0.003	0.011	-0.019	0.025
	PT → AS	0.070	0.012	0.047	0.093
	DS → AS	0.540	0.047	0.449	0.631
	Age → AS	-0.149	0.072	-0.290	-0.009
Model B3F	CPA → PT	0.683	0.040	0.607	0.763
	Age → PT	0.232	0.368	-0.479	0.955

CPA → DS	0.104	0.012	0.081	0.127
PT → DS	0.148	0.008	0.132	0.163
Age → DS	0.225	0.074	0.077	0.370
CPA → AS	0.003	0.010	-0.016	0.022
PT → AS	0.066	0.009	0.048	0.082
DS → AS	0.577	0.037	0.502	0.648
Age → AS	-0.051	0.059	-0.163	0.067

Note. M: Male adolescents, N = 806; F: Female adolescents, N = 797.

CPA: Childhood Psychological Abuse; PT: Psychological Trauma; AS: Anxiety Symptoms; DS: Depressive Symptoms.

Model A3M & Model A3F: Chain mediation model, controlling for age; Model B3M & Model B3F: Chain mediation model, controlling for age.

Bootstrap = 5000.
